# Influence of adverse drug events on the mortality and the length of hospital stay in ICUs in Japan: the JADE Study

**DOI:** 10.1186/cc12448

**Published:** 2013-03-19

**Authors:** Y Ohta, M Sakuma, D Bates, T Morimoto

**Affiliations:** 1 Kinki University, Osaka, Japan; 2 Harvard University, Boston, MA, USA

## Introduction

Adverse drug events (ADEs) are associated with a substantial increase in morbidity and mortality in any setting. Because patients in ICUs were critically ill with complex diseases and varied organ dysfunction, the incidence of ADEs on such patients is much more crucial than the counterparts. We thus assessed the nature of ADEs and their influence in ICUs.

## Methods

We conducted a prospective cohort study at ICUs at three large tertiary-care hospitals in Japan. Trained research nurses reviewed all medical charts, incident reports and reconciliations from the pharmacy to identify suspected ADEs as well as the background of patients. ADEs are any injuries that result from the use of a drug. After suspected ADEs are collected by research nurses, physician reviewers independently evaluated them and classified them as ADEs or rule violations. We used the validated methodology [1].

## Results

We included 459 patients with 3,231 patient-days. The median age was 70 years and the median length of stay was 3 days. In total, 70 patients (15%) had at least one ADE during their stay in the ICU. The median ICU stay in patients who had ADEs was 14 days while 2 days in patients who had no ADEs (*P *0.0001). The median length of the ADE onset days since admission was 3 days. Regarding the mortality, 73 patients (16%) were dead during their ICU stay: 12 deaths (17%) in patients who had ADEs and three of 12 deaths were caused by an ADE, and 61 deaths (16%) in counterparts (*P *= 0.8). There were no significant differences of patients' characteristics between patients with ADEs and without ADEs (Table 1).

**Table 1:** Patient characteristics

	Patients	Patients	
	with	without	
	ADEs	ADEs	
Characteristic	(*n *= 70)	(*n *= 389)	*P *value
Age ≥65 years, *n *(%)	49 (70)	227 (58)	0.08
Male, *n *(%)	40 (57)	250 (64)	0.2
Department			
Medicine	35 (50)	228 (59)	0
Surgery	35 (50)	161 (41)	0.2
Unconsciousness, GCS ≤8	13 (19)	91 (23)	0.4
Heart failure, NYHA = 4	37 (53)	216 (56)	0.7
Respiratory failure	44 (62)	262 (67)	0.5
Kidney failure	6 (9)	58 (15)	0.2
Clinical laboratory measurements, median (25%, 75%)			
Blood urea nitrogen (mg/dl)	21 (15, 39)	19 (14, 31)	0.2
Creatinine (mg/dl)	0.8 (0.5, 1.5)	0.9 (0.7, 1.7)	0.8
Aspartate aminotransferase (IU/l)	27 (20, 47)	30 (22, 48)	0.5
Alanine transaminase (IU/l)	20 (13, 42)	20 (12, 33)	1.0
Total protein (g/dl)	6.7 (5.9, 7.4)	6.6 (5.9, 7.3)	0.8
Number of medications on admission ≥4,	38 (54)	223 (57)	0.6
*n *(%)			
Number of medications on admission,	5 (3, 7)	5 (4, 8)	0.7
median (25%, 75%)			

**GCS, Glasgow Common Scale; NYHA, New York Heart Association**.

## Conclusion

ADEs were associated with longer stay and caused a part of death in ICU (4%) although they did not increase the mortality. Because the characteristics of patients were not associated with ADEs, early detection and intervention for ADEs could be important to improve the morbidity and reduce the death caused by ADEs in ICUs.
